# Alterations in Hypothalamus-Pituitary-Adrenal/Thyroid Axes and Gonadotropin-Releasing Hormone in the Patients with Primary Insomnia: A Clinical Research

**DOI:** 10.1371/journal.pone.0071065

**Published:** 2013-08-09

**Authors:** Lan Xia, Gui-Hai Chen, Zhi-Hua Li, Song Jiang, Jianhua Shen

**Affiliations:** 1 Department of Neurology, the First Affiliated Hospital of Anhui Medical University, Hefei, People’s Republic of China; 2 Department of Neurology, the Second Affiliated Hospital of Anhui Medical University, Hefei, People’s Republic of China; 3 Sleep Research Laboratory, Department of Psychiatry, University Health Network, University of Toronto, Toronto, Canada; John Hopkins University School of Medicine, United States of America

## Abstract

The hypothalamus-pituitary-target gland axis is thought to be linked with insomnia, yet there has been a lack of further systematic studies to prove this. This study included 30 patients with primary insomnia (PI), 30 patients with depression-comorbid insomnia (DCI), and 30 healthy controls for exploring the alterations in the hypothalamus-pituitary-adrenal/thyroid axes’ hormones and gonadotropin-releasing hormone (GnRH). The Pittsburgh Sleep Quality Index was used to evaluate sleep quality in all subjects. The serum concentrations of corticotrophin-releasing hormone (CRH), thyrotrophin-releasing hormone (TRH), GnRH, adrenocorticotropic hormone (ACTH), thyroid stimulating hormone (TSH), cortisol, total triiodothyronine (TT3), and total thyroxine (TT4) in the morning (between 0730 h and 0800 h) were detected. Compared to the controls, all hormonal levels were elevated in the insomniacs, except ACTH and TSH in the PI group. Compared to the DCI patients, the PI patients had higher levels of CRH, cortisol, TT3, and TT4 but lower levels of TRH, GnRH, and ACTH. Spearman’s correlation analysis indicated that CRH, TRH, GnRH, TSH, cortisol, TT4, and TT3 were positively correlated with the severity of insomnia. The linear regression analysis showed that only CRH, GnRH, cortisol, and TT3 were affected by the PSQI scores among all subjects, and only CRH was included in the regression model by the “stepwise” method in the insomnia patients. Our results indicated that PI patients may have over-activity of the hypothalamus-pituitary-adrenal/thyroid axes and an elevated level of GnRH in the morning.

## Introduction

Insomnia is defined as a disorder characterized by difficulty in initiating and/or maintaining sleep, even when a person has enough time, adequate opportunity, and circumstances for sleep. Insomnia also includes subjective reports of daytime symptoms, such as fatigue, mood disturbances, cognitive impairments, and working-related problems [1]–[3].

Primary insomnia (PI) occurs in the absence of mental, medical, neurological, or drug use or abuse causes or any other sleep disorders [4]. Although insomnia has been studied intensively for several decades, the underlying mechanism of PI remains to be determined. The hyper-arousal hypothesis of PI, which indicates that the over-activity of the hypothalamus-pituitary-adrenal (HPA) axis is linked to insomniac hyper-arousal [2], [3], [5], [6], has been put forward for 15 years [7]. However, the supportive evidence is insufficient, and the existing results are inconsistent [8]–[12]. The heterogeneity of samples (e.g. the source and severity of insomnia), small sample sizes (most contained fewer than 15 subjects) and different measurements (e.g. blood or urine test) may be the reasons for the conflicting results. Therefore, the exact alterations in the HPA axis in PI patients are still under investigation.

Current data have suggested that the hypothalamus-pituitary-thyroid (HPT) and hypothalamus-pituitary-gonadal (HPG) axes may also participate in the onset and development of insomnia. Patients with hyperthyroidism or hypothyroidism experience sleep disturbances, such as difficulty initiating and maintaining sleep or reduced or even increased slow wave sleep [13]. Intravenously administered thyrotrophin-releasing hormone (TRH) can alter sleep parameters, such as reduced non-rapid eye movement sleep and sleep efficiency or increased wakefulness [14]. In terms of the HPG axis, studies have shown that postmenopausal women with lower estradiol and higher luteinizing hormone levels had poor sleep quality, such as decreased slow wave sleep and increased rapid eye movement densities [13]. After hormone replacement therapy, such as oral progesterone [15] or a skin patch estradiol supplement [16], intermittent wakefulness reduction and rapid eye movement sleep improvement were observed in postmenopausal women. Nevertheless, scarce evidence of alterations in the HPT and HPG axes has been obtained directly from PI patients.

Unlike PI, alterations in the HPA and HPT axes in depressive patients have been intensively studied, and the results are relatively consistent. Several lines of evidence indicate the importance of the HPA axis in depression. Over-activity in the HPA axis is found in many people with depression, represented in increased levels of corticotrophin-releasing hormone (CRH), adrenocorticotropic hormone (ACTH), and cortisol [17] and enlarged adrenal glands [18]. Studies have shown that patients with hypothyroidism may develop mild to severe depression [19] and that patients with major depression may have clinical or subclinical hypothyroidism with characteristic alterations in triiodothyronine, thyroxine, thyroid stimulating hormone (TSH), and TRH levels [18]. Depressive male patients may have low testosterone [20], and menopausal women may have depressed mood [21]. Because of the close relationship between depression and insomnia, taking depression-comorbid insomnia (DCI) as a positive control will aid in understanding the mechanism underlying PI.

We hypothesized that PI patients have similar alterations to the DCI patients in the HPA/HPT/HPG axes. Therefore, in the present study, we detected the serum levels of CRH, ACTH, cortisol, TRH, TSH, total triiodothyronine (TT3), and total thyroxine (TT4) with strict inclusion criteria and clinic sample. Meanwhile, because of the complex effects of age and gender, we only examined gonadotropin-releasing hormone (GnRH) in the HPG axis.

## Materials and Methods

### Subjects

Sixty insomnia outpatients (19 men, 41 women) were obtained from the Clinic of Sleep and Memory Disorder at the First Affiliated Hospital of Anhui Medical University. The patients, who had received at least nine years of education, were diagnosed by a senior clinician, who is a specialist in sleep medicine, according to the *Diagnostic and Statistical Manual of Mental Disorders*, Fourth Edition, text revision (DSM-IV-TR) criteria [4] and categorized into PI (n = 30) and DCI (n = 30, positive controls) groups. Additionally, the PI patients should have suffered from insomnia for six months or over. Subjects with bipolar disorder, thyroid diseases, Sheehan syndrome, obstructive sleep apnea syndrome, restless legs syndrome, medical diseases, or any other psychiatric disorders were excluded. None of the subjects took drugs that would potentially interfere with sleep, mood, or endocrine function during the last three months before inclusion. Pregnant women were also ruled out. Thirty demographically similar healthy subjects who accompanied the patients or had a medical examination were selected as negative controls. These subjects had no subjective complaint of insomnia and mood disorder with a Pittsburgh Sleep Quality Index (PSQI) score <7 [22], a 17-item Hamilton Depression Rating Scale (HAMD-17) score <7 [22], and a Chinese-Beijing Version of Montreal Cognitive Assessment (MoCA-C) score ≥26 (www.mocatest.org), which indicated good sleep and mood and normal cognitive function. The study was done with permission from the Clinical Trial Ethics Committee of the First Affiliated Hospital of Anhui Medical University and the written consent of the subjects.

### Baseline Data Collection

Baseline data, including demographic characteristics (i.e., age, gender, and educational information), medical history, and family history were collected using a researcher-developed questionnaire.

### Assessment of Insomnia, Depressive Mood, and Cognitive Dysfunction

Severity of insomnia and depression were assessed by a well-trained clinician using PSQI and HAMD-17, respectively. In China, a PSQI score ≥7 has high diagnostic sensitivity and specificity in distinguishing patients with sleep problems from normal subjects [22]. A HAMD-17 score ≥17 suggests moderate to severe depression [22]. Regarding the score of MoCA-C, used to assess the general cognitive function in this study, ≥26 is considered normal (www.mocatest.org).

### Blood Sample Collection and Hormone Tests

Blood samples were collected in the morning (between 0730 h and 0800 h) in a sitting position after resting and relaxing for 30 minutes without heavy exercise and were separated immediately by centrifugation. The obtained sera were stored at –80°C until hormonal assaying. The serum concentrations of hypothalamic releasing hormones (CRH, TRH, and GnRH), pituitary hormones (ACTH and TSH), and target gland hormones (cortisol, TT3, and TT4) were examined in all subjects using the ELISA method (Microplate reader, Labsystems Multiskan MS 352, USA; Wellwasher, Thermo Labsystems AC8, USA; ELISA reagents, Shanghai Jianglai Co., Ltd., China), with sensitivity >84% and specificity >98%, respectively. All sample measurements were run in duplicate, and the averages were calculated for analysis. Intra- and inter-assay coefficients of variation were less than 9% and 11%, respectively.

### Statistical Analysis

The data were explored to reveal their feature of distribution before statistical tests were performed. When the distribution of data was normal, the variables were expressed as means (standard deviation [SD]), and one-way analysis of variance (ANOVA) was chosen to make a pairwise comparison in the parameter test. When the distribution of data was non-normal, the variables were expressed as the 50th quartile (25^th^ and 75th quartile) (P_50_ [P_25_, P_75_]), and the Kruskal-Wallis *H* test was used with a pairwise comparison conducted by manual calculation [23]. Correlations between the serum hormones and PSQI scores were analyzed using Spearman’s correlation analysis. To investigate the effects of hormones on insomnia, linear regression analysis was used. To compare the diagnostic information provided by different biochemical markers, receiver operating characteristics (ROC) analysis was used according to the calculated area under the curve (AUC), optimal cut-off, sensitivity, and specificity. The AUCs at the levels of 0.5–0.7, 0.7–0.9, and >0.9 indicated that the biochemical markers had low, moderate, and high diagnostic values, respectively [24]. All the data were analyzed using SPSS 13.0 for Windows. The level of statistical significance was set at a two-tailed *P*-value of 0.05.

## Results

### Background Data

Table 1 shows the detailed background information of each group. There were no significant differences in gender, age, educational level, and MoCA-C score among the three groups (*P*s >0.05), indicating that the cases included in this study were well matched and controlled. Compared to the controls, the average PSQI and HAMD-17 scores were significantly higher in the insomniacs (*P*s <0.001). The difference in PSQI scores between the two insomnia groups was insignificant (*P*>0.05), but the average HAMD-17 score in the PI group was significantly lower than that in the DCI group (*Ps* <0.05).

**Table 1:** Demographic characteristic of insomnia patients and controls.

Items		Controls	PI	DCI	Statistic	*P-*value
Numbers		30	30	30		
Gender (male/female)		10/20	9/21	10/20	*χ* ^2^ = 0.10	0.95
Age (yr)	Mean (SD)	37.8 (11.2)	42.4 (10.5)	43.0 (10.6)	*F* = 2.10	0.13
	Range	21–65	23– 62	22– 64		
Education (yr)		12.0 (9.0, 15.3)	12.0 (9.0, 15.0)	12.0 (9.0, 15.0)	*H* = 0.48	0.79
PSQI (score)		2.0 (1.0, 4.0)	17.5 (15.0, 19.0)*	16.0 (15.0, 18.0)*	*H* = 60.47	<0.001
HAMD-17 (score)		3.8 (2.8)	12.7 (3.5)* #	20.7 (4.4)*	*F* = 162.42	<0.001
MoCA-C (score)		26.8 (1.5)	27.0 (24.5, 28.0)	25.0 (1.8)	*H* = 5.80	0.06

Abbreviations: PI, primary insomnia; DCI, depression-comorbid insomnia; PSQI, Pittsburgh Sleep Quality Index; HAMD-17, Hamilton Depression Rating Scale (17 items); MoCA-C, Chinese-Beijing Version of Montreal Cognitive Assessment.

Expression: normal distribution variables (Mean [SD]); nonnormal distribution variables (P_50_ [P_25_, P_75_]).

* *P*<0.05, Compared to controls.

# *P*<0.05, Compared to DCI group.

### Alterations in HPA and HPT Axes Hormones and GnRH

The hormonal data are displayed in Table 2. The serum levels of CRH, TRH, cortisol, TT3, and TT4 were significantly higher in both insomnia groups compared to the control group (*P*s <0.001). However, the levels of ACTH and TSH were higher only in the DCI patients (*P*s <0.05), not in the PI patients (*Ps* >0.05). Between insomnia groups, the PI patients had higher levels of CRH, cortisol, TT3, and TT4 (*P*s <0.05) but lower levels of TRH and ACTH (*P*s <0.05) and comparable TSH levels (*P*>0.05). The GnRH level was significantly higher in both insomnia groups relative to the control group (*Ps* <0.001), as well as in the DCI group relative to the PI group (*P*<0.001).

**Table 2:** Serum levels of hormones in patients with insomnia and controls.

Axes	Terms	Controls	PI	DCI	Statistic	*P-*value
HPA	CRH (ng/l)	65.4 (27.8)	126.7 (103.4, 180.6)* #	102.6 (86.3, 138.8)*	*H* = 38.65	<0.001
	ACTH (ng/l)	73.4 (5.9)	77. 6 (10.2)#	91.5 (86.6, 93.6)*	*H* = 24.71	<0.001
	Cortisol (µg/l)	126.7 (11.8)	230.3 (12.1)* #	198.3 (21.1)*	*F* = 347.04	<0.001
HPT	TRH (ng/l)	82.7 (6.8)	97.7 (4.5)* #	133.6 (5.4)*	*F* = 640.41	<0.001
	TSH (mIU/l)	2.5 (0.4)	2.6 (0.3)	2.8 (0.2)*	*F* = 6.95	0.002
	TT3 (µg/l)	2.6 (0.2)	4.7 (0.2)* #	4.0 (0.5)*	*F* = 326.54	<0.001
	TT4 (µg/l)	133.8 (14.6)	240.8 (234.8, 246.5)* #	198.7 (20.9)*	*H* = 254.70	<0.001
HPG	GnRH (ng/l)	18.3 (1.0)	24.1 (1.0)* #	28.6 (1.0)*	*F* = 790.81	<0.001

Abbreviations: PI, primary insomnia; DCI, depression-comorbid insomnia; HPA, Hypothalamus-pituitary-adrenal; HPT, Hypothalamus-pituitary-thyroid; HPG, Hypothalamus-pituitary-gonads; CRH, corticotrophin-releasing hormone; ACTH, adenocorticotropic hormone; TRH, thyrotrophin-releasing hormone; TSH, thyroid stimulating hormone; TT3, total triiodothyronine; TT4, total thyroxine; GnRH, gonadotropin-releasing hormone.

Expression: normal distribution variables (Means [SD]); nonnormal distribution variables (P_50_ [P_25_, P_75_]).

* *P*<0.05, Denotes a significant difference compared to controls;

# *P*<0.05, Compared to DCI group.

### Associations between the Severity of Insomnia and the Serum Levels of Hormones

The Spearman’s correlation analysis indicated that the levels of CRH, TRH, GnRH, TSH, cortisol, TT3, and TT4 were significantly positively correlated with PSQI scores, respectively (*P*s <0.05), in all subjects but significantly positively and negatively associated with the CRH and ACTH levels, respectively, in the insomniacs without the controls (*P*s <0.05). Then, the linear regression model was used to analyze the association between the severity of insomnia and hormonal serum levels using the “enter” method with all requested variables (CRH, ACTH, cortisol, TRH, TSH, TT3, TT4, and GnRH) entered. According to the linear regression analysis, CRH, GnRH, cortisol, and TT3 were associated with PSQI in all subjects (*P*s <0.05), but only CRH was included in the regression model by the “stepwise” method in all insomnia patients (Beta = 0.389, *P*<0.01). However, after separating all subjects into the PI, DCI, and control groups, no association between the PSQI score and the hormonal serum levels was found in each group (*P*s >0.05). These results are presented in Table 3.

**Table 3:** Correlation and regression analysis for serum levels of hormones and PSQI scores.

Subjects	Methods	Statistic	CRH	ACTH	Cortisol	TRH	TSH	TT3	TT4	GnRH
All Subjects	Spearman’s Correlation	r	0.659**	–0.051	0.736**	0.573**	0.214*	0.714**	0.648**	0.655**
	Linear Regression^a^	Beta	0.175**	–0.043	0.212*	–0.079	0.021	0.315**	–0.039	0.486**
Insomniacs	Spearman’s Correlation	r	0.376*	–0.306*	0.228	–0.239	−0.059	0.135	–0.010	–0.086
	Linear Regression^a^	Beta	0.389**	–0.129	0.023	–0.491	0.054	0.0118	–0.322	0.334

Abbreviations: PI, primary insomnia; DCI, depression-comorbid insomnia; PSQI, Pittsburgh Sleep Quality Index; CRH, corticotrophin-releasing hormone; ACTH, adenocorticotropic hormone; TRH, thyrotrophin-releasing hormone; TSH, thyroid stimulating hormone; TT3, total triiodothyronine; TT4, total thyroxine; GnRH, gonadotropin-releasing hormone.

a All requested variables (CRH, ACTH, cortisol, TRH, TSH, TT3, TT4 and GnRH) entered.

* *P*<0.05;

** *P*<0.01.

### Suitability of Blood Hormones as Diagnostic Biomarkers for PI

The results of the ROC analysis with values for sensitivity, specificity and the AUC are listed in Table 4. The AUCs of CRH, cortisol, TRH, TT3, TT4, and GnRH were larger than 0.90, respectively, which were good for predicting PI compared to the controls. The optimal cut-off values for them were 89.7 ng/l, 170.9 µg/l, 90.0 ng/l, 3.7 µg/l, 190.4 µg/l, and 21.8 ng/l, respectively. These values suggested that the patients should be considered as having PI when they had the hormonal levels over the corresponding cut-off values. Furthermore, the AUCs of cortisol, TRH, TT3, TT4, and GnRH were larger than 0.90, respectively, which performed well in predicting PI compared to the DCI group. The optimal cut-off values were 218.8 µg/l, 111.8 ng/l, 4.4 µg/l, 227.6 µg/l, and 26.5 ng/l, respectively. According to the levels of hormones in the two insomnia groups, the diagnosis of PI was preferentially considered when the levels of cortisol, TT3, and TT4 were higher than their cut-off values, while the diagnosis of DCI was preferentially considered when the levels of TRH and GnRH were higher than their cut-off values.

**Table 4:** Characteristics of potential blood biomarkers for PI in ROC analysis.

	PI compared to Controls				PI compared to DCI			
Hormones	AUC (95%CI)	Cut-off	Sensitivity	Specificity	AUC (95%CI)	Cut-off	Sensitivity	Specificity
CRH (ng/l)	0.94 (0.84–0.99)	≥89.7	0.90	0.90	0.66 (0.52–0.80)	≥101.7	0.83	0.50
ACTH (ng/l)	0.68 (0.54–0.82)	≤81. 5	0.70	0.67	0.89 (0.81–0.97)	≤83.0	0.87	0.77
Cortisol (µg/l)	1.00 (1.00–1.00)	≥170.9	1	1	0.92 (0.84–0.99)	≥218.8	0.90	0.89
TRH (ng/l)	0.97 (0.94–1.00)	≥90.0	1	0.87	1.00 (1.00–1.00)	≤111.8	1	1
TSH (mIU/l)	0.52 (0.38–0.67)	≥2.3	0.87	0.30	0.75 (0.62–0.88)	≤2.6	0.87	0.60
TT3 (µg/l)	1.00 (1.00–1.00)	≥3.7	1	1	0.91 (0.824–1.00)	≥4.4	1	0.86
TT4 (µg/l)	1.00 (1.00–1.00)	≥190.4	1	1	0.97 (0.93–1.00)	≥227.6	0.93	0.96
GnRH (ng/l)	1.00 (1.00–1.00)	≥21.8	1	1	1.00 (1.00–1.00)	≤26.5	1	1

Abbreviations: PI, primary insomnia; DCI, depression-comorbid insomnia; AUC, Area under the receiver operating curve; CI, confidence interval. CRH, corticotrophin-releasing hormone; ACTH, adenocorticotropic hormone; TRH, thyrotrophin-releasing hormone; TSH, thyroid stimulating hormone; TT3, total triiodothyronine; TT4, total thyroxine; GnRH, gonadotropin-releasing hormone.

## Discussion

We simultaneously explored the alterations in the neuroendocrine system in the HPA and HPT axes and GnRH at multiple levels (i.e., the hypothalamus, pituitary, and target gland levels) in the PI and DCI patients. The current study mainly indicated that: (1) in the morning, the insomniacs had higher levels of all serum hormones except for ACTH and TSH in the PI group, and the PI patients had higher CRH, cortisol, TT3, and TT4 and lower ACTH, TRH, and GnRH than the DCI patients; (2) higher levels of all these hormones, except ACTH, were associated with more severe insomnia, but only CRH affected insomnia independently; and (3) the AUCs of cortisol, TT3, TT4, and GnRH were larger than 0.90 (i.e. high diagnostic value) compared to the controls and DCI patients.

Although growing literature on insomnia has been published, the initiative and sustaining mechanism of PI remains obscure. Many studies have indicated that the neuroendocrine system is involved in sleep regulation, including a lot of hormones [14], [25], [26]. Nevertheless, very little is known about the effects of these hormones on insomnia, let alone PI.

For the HPA axis in PI patients, only a few studies have been reported with inconsistent results. Some studies have indicated that insomniacs tended to have higher levels of plasma ACTH and urinary/plasma cortisol [9], [11], while others have found little difference between insomniacs and controls with respect to urinary/serum cortisol [8], [10] and plasma ACTH/cortisol after a dexamethasone/CRH test [12]. Obviously, the available data on the HPA axis in PI patients referred only to ACTH and cortisol, and there is a lack of studies on CRH. The different sample sizes, subject source, and severity of disease might be related to the discrepancies in the published findings. In this study, we enlarged the case numbers and enrolled insomniacs who met the DSM-IV-TR diagnosis criteria from the clinic setting. We also assessed the subjects’ insomnia and depression severity and compared the PI patients with positive (i.e. DCI) and negative (i.e. healthy) controls simultaneously. Our results showed that, compared to the controls, serum levels of CRH, ACTH, and cortisol in the morning were elevated in the insomniacs suffering from PI or DCI. Interestingly, the PI patients with moderate to severe insomnia in our study had higher levels of CRH and cortisol than the DCI patients, suggesting that PI-type insomnia may be more closely associated with enhanced HPA-axis activity relative to DCI-type insomnia [2], [3], [27]. Furthermore, DCI-type insomniacs, rather than PI-type insomniacs, had increased ACTH level, which was consistent with previous results for depressive patients [14], [27], [28].

To our knowledge, there have been no studies on the alteration of the HPT and HPG axes in PI patients. Currently, a causal link between depression and HPT axis is suspected. Available data show that the levels of TSH, TT3, and TT4 might be slightly increased or decreased, and the levels of free thyroxine might increase in different types of patients with depressive disorder [29]–[32]. In our study, patients diagnosed with a depressive disorder and moderate to severe insomnia had elevated levels of TRH, TSH, TT3, and TT4 compared to the controls, as did the PI patients. Surprisingly, the PI patients had higher TT3 and TT4 in the morning but lower TRH and TSH than the DCI patients. Considering the regulation of the HPT axis [33], our results suggested that more over-activity of the HPT axis took place under the condition of PI relative to DCI. By reason of the complex effects of age and gender, we examined only GnRH in the HPG axis. The present results showed that both groups of insomniacs had higher GnRH levels in the morning than the controls, which provided support for the notion that the HPG axis was associated with sleep and mood regulation [14], [21]. Between the two insomnia groups, the GnRH level was lower in the PI patients than in the DCI patients. Further studies need to be carried out to completely understand the variations of the HPG axis in the PI patients.

Our results showed that CRH, TRH, GnRH, TSH, cortisol, TT3, and TT4 had positive correlations with the severity of insomnia, suggesting the more severe insomnia was, the more active the HPA, HPT, and HPG axes were. For the most possible factors exerted on insomnia among the sleep-related hormones mentioned above, our linear regression analysis indicated that only CRH, GnRH, cortisol and TT3 were closely associated with PSQI scores in all subjects, but only CRH was included in the regression model with the insomniacs. In consistent with previous studies [12], [27], [34], our data suggested that in the HPA axis, CRH may play a more important role in insomnia. Moreover, our results also showed that the PI patients had higher activity of HPA axis and lower activity of HPT and HPG axes than the DCI patients. As indicated by previous data, an abnormal positive feedback effect on CRH release occurs in chronic insomniacs [27], [34]–[36]. So that, the over-activity of HPT and HPG axes was partially suppressed by the dominant hyperactivity of the HPA axis [36]–[44] in the PI patients.

Because of the differences of serum hormonal levels among the patients with PI, DCI and the controls, we used the ROC analysis to calculate the cut-off values of different hormones to discriminate between a true and a false PI diagnosis as established by the DSM-IV-TR criteria. The results showed that the AUCs of CRH, cortisol, GnRH, TRH, TT3, and TT4 were larger than 0.9, indicating that the optimal cut-off values with both high sensitivity and specificity simultaneously (Table 4) among the three groups may provide strong cues for considering PI. We speculated that these hormones might act as diagnostic and differential diagnostic objective markers of PI. Cortisol, TRH, TT3, TT4, and GnRH distinguished PI well among the healthy and DCI patients, while CRH acted only between the PI and the normal participants.

There were a few of limitations in this study. First, we evaluated the sleep condition using only rating scales, which lacked objective criteria. Second, we examined only one time point of the hormonal serum levels, which cannot present all the alterations in the rhythms of the hypothalamus-pituitary-target gland axes related to hormones, and the peripheral serum levels could not thoroughly reflect the hormone concentrations in the central nervous system. Third, in order to reflect the sleep condition of subjects at home more authentically, we restrained the sample collection time and condition relatively to increase the comparability as more as possible. Although blood samples were collected between 0730 h and 0800 h, a relatively short and fixed time, the wake time was not controlled, which is a major issue in some hormones analyses e.g. cortisol. The concentration might fluctuate very quickly in the first hour after awakening and increase difficulty of comparing the concentration among participants. Fourth, because of the strict inclusion criteria and the practical medical condition in the study area, the sample size for the PI patients was limited, and we did not distinguish the subtype of PI according to the International Classification of Sleep Disorders, Second Edition [1]. Moreover, we did not distinguish whether the patients’ condition was aggravated in the short time when they came to see a doctor, which might affect the results. Considering these limitations, further studies are warranted.

The current study was the first to systematically investigate alterations in the HPA and HPT axes and GnRH in PI and DCI patients. We found that PI patients had over-activity of the HPA and HPT axes and elevated levels of GnRH in the morning. The activity of the HPA axis was closely associated with the sensitivity of insomnia. Cortisol, TT3, TT4, GnRH, and CRH might be considered somewhat useful as diagnostic and differential diagnostic markers for PI.
